# Validation of a Machine Learning Approach to the Analysis of Multifocal Electroretinograms for Hydroxychloroquine Retinopathy

**DOI:** 10.1167/tvst.15.7.29

**Published:** 2026-07-28

**Authors:** Godfrey Wong, Gareth Mercer, Brian G. Ballios, Tom Wright

**Affiliations:** 1 Institute of Medical Science, Temerty Faculty of Medicine, University of Toronto, Toronto, Ontario, Canada; 2 Kensington Vision and Research Centre, Toronto, Ontario, Canada; 3 Department of Ophthalmology, St. Michael's Hospital, Unity Health, Toronto, Ontario, Canada; 4 Department of Ophthalmology and Vision Sciences, Temerty Faculty of Medicine, University of Toronto, Toronto, Ontario, Canada; 5 Donald K. Johnson Eye Institute, University Health Network, Toronto, Ontario, Canada

**Keywords:** hydroxychloroquine (HCQ), multifocal electroretinogram (mfERG), screening guideline, machine learning, external validation

## Abstract

**Purpose:**

Hydroxychloroquine (HCQ) retinopathy is detectable through multimodal ophthalmic screening, yet individual diagnostic tests each have inherent limitations. Machine learning may simplify monitoring, but few HCQ screening algorithms have undergone rigorous external validation. This study evaluated the clinical utility of the Multifocal Electroretinogram Classification Interface (MERCI) algorithm by assessing its ability to predict HCQ retinopathy compared with diagnoses derived from American Academy of Ophthalmology (AAO) guidelines.

**Methods:**

Patients referred for HCQ toxicity screening underwent perimetry, spectral-domain optical coherence tomography, fundus autofluorescence photography, and multifocal electroretinogram (mfERG) testing. Performance was assessed in a temporal dataset (participants overlapping with the development dataset) and a novel dataset (newly enrolled participants). Reference diagnoses were assigned using AAO guideline-based decision trees informed by ancillary tests results. MERCI generated corresponding mfERG-based predictions, which were compared with reference diagnoses to compute performance metrics for each dataset.

**Results:**

Using the decision trees, the temporal (*n* = 145) and novel (*n* = 300) datasets showed retinopathy prevalences of 12.4% and 9.7%, respectively. MERCI yielded 63/145 (43.4%) and 115/300 (38.3%) false-positive cases, respectively. MERCI achieved strong sensitivity (1.000 temporal; 0.931 novel) and negative predictive value (NPV) (1.000 temporal; 0.987 novel), with comparable area under the curve between datasets (0.759 temporal; 0.805 novel).

**Conclusions:**

MERCI retained high sensitivity and NPV when validated against temporally distinct cohorts using a clinical definition of HCQ toxicity. This suggests MERCI's potential as a screening tool for HCQ retinopathy.

**Translational Relevance:**

The mfERG-based machine learning models may improve screening efficiency and clinical decision-making for HCQ users.

## Introduction

Hydroxychloroquine (HCQ) is a disease-modifying antirheumatic drug widely prescribed for chronic inflammatory diseases such as systemic lupus erythematosus (SLE) and rheumatoid arthritis (RA) due to its favorable systemic safety profile and long-term efficacy.^1^ Despite its therapeutic benefits, chronic HCQ use carries a risk of retinal toxicity. HCQ retinopathy manifests as photoreceptor outer segment loss followed by retinal pigment epithelial damage in more severe cases, typically in the parafoveal region. Retinopathy is progressive, irreversible and can lead to loss of central vision. Prevalence was once thought to be rare at only approximately 0.5%, because toxicity was only detected at its most severe stage, the clinical “bull's-eye maculopathy.”^2^^,^^3^ With the rise of more sensitive diagnostic technology, retinal changes that were subtle or asymptomatic were being identified at earlier timepoints. As such, a higher volume of cases was observed. Prevalence was then estimated to be at 7.5% after five years and increasing to 20% to 50% after 20 years.^4^ Additional factors (i.e., dosage greater than 5.0 mg/kg of real body weight, kidney disease, and concurrent tamoxifen use) have been identified to increase the risk of retinopathy.^4^ Ophthalmological guidelines by the American Academy of Ophthalmology (AAO) and the Royal College of Ophthalmology (RCOphth) were designed to emphasize the importance of regular, multimodal screening to detect toxicity before significant visual impairment occurs.^5^^,^^6^

Screening guidelines specify diagnostic tests that are effective in HCQ retinopathy monitoring. Visual field testing (VF), particularly 10-2 static perimetry, assesses central visual sensitivity and can reveal early paracentral scotomas. Spectral-domain optical coherence tomography (SD-OCT) offers high-resolution imaging of the macula, enabling clinicians to detect thinning of the outer retina, parafoveal ellipsoid zone disruption, or the characteristic “flying saucer” sign.^7^ Fundus autofluorescence photography (FAF) visualizes lipofuscin distribution in the retinal pigment epithelium, highlighting areas of stress or cellular damage through abnormal autofluorescence patterns. Multifocal electroretinography (mfERG) measures localized retinal electrical responses to light stimulation, allowing functional assessment of photoreceptor and bipolar cell activity across the central retina. The stimulus is arranged as a central hexagon with four surrounding concentric rings of hexagonal elements, each scaled to ensure uniform retinal response contribution. A ring ratio is calculated as the amplitude of the central hexagon divided by the average amplitude of a parafoveal ring.^8^ In HCQ toxicity, parafoveal dysfunction can cause a decrease in any of the average ring amplitudes, but particularly in the two rings closest to the central ring, thus elevating ring ratios above thresholds.^8^

Each diagnostic test has its own limitations. The 2016 AAO recommendations suggest the results of a subjective test should be corroborated by an objective modality.^5^ However, these guidelines lack clarity regarding which of the three objective tests should be prioritized in the screening process as the FAF, OCT, and mfERG do not have equal sensitivities. Moreover, VF—which is the only subjective test—is highly variable. One study found that 33.1% of visual fields were unreliable, and 24.9% were of poor quality.^9^ Although mfERG is highly sensitive, its practicality as an HCQ screening tool is hindered by the small number of clinicians who are trained to analyze and interpret the findings.

Advances in computational ophthalmology have opened new opportunities for automated detection of retinal disease, particularly through machine learning. Machine learning models have been used to diagnose diabetic retinopathy, retinopathy of prematurity, and age-related macular degeneration.^10^^–^^12^ Presently, there are few machine learning algorithms that exist for HCQ toxicity screening, and even fewer so that have been externally and temporally validated.^13^^–^^17^ Temporal validation determines whether outcomes of a prognostic model are reproducible over time, and external validation determines if the algorithm is generalizable to novel data.

A machine learning algorithm, the Multifocal Electroretinogram Classification Interface (MERCI), was developed to automatically identify HCQ toxicity using ring ratios as the reference standard.^18^ A support vector machine (SVM) was chosen as the machine learning model because of its ability to handle multiple features, remain robust to outliers, and segregate using non-linear boundaries. In MERCI, each of the 100 SVM classifiers take the overall signal strength of the mfERG, ring variation, and the ring ratios into account to characterize whether the mfERG is abnormal or not. By summing up the total number of classifiers that label the mfERG as abnormal, a MERCI score is assigned to the mfERG which represents the likelihood of HCQ retinopathy. An internal validation of the model found that it could distinguish eyes with HCQ retinopathy with a sensitivity of 90.9% and a specificity of 84.0%.^18^ Although MERCI shows promise in identifying toxicity in independent tests, we aimed to assess whether it could achieve accuracy comparable to a clinical diagnosis based on the 2016 AAO HCQ screening guidelines. In this study, we investigated MERCI's clinical utility and hypothesized that its external and temporal validation would yield performance metrics that were robust.

## Methods

### Ethics

This validation study was approved by the University of Toronto Human Research Ethics Board. Written informed consent was obtained from all participants. All aspects of the study complied with the tenets in the Declaration of Helsinki.

### Participants and Data Collection

Patients referred to the electrophysiology clinic at the Kensington Vision and Research Centre (KVRC) between July 23, 2024, to December 19, 2025, for HCQ toxicity screening were invited to participate. As part of their routine clinical care, all four ancillary tests recommended by the AAO were performed binocularly on each participant. For individuals with multiple visits during the study period, data from the most recent visit were used for analysis.

#### Tests and Procedures Used

##### Perimetry

Automated visual field testing was performed using the SITA 10-2 protocol on the Humphrey Field Analyzer 3 (Carl Zeiss Meditech AG, Jena, Germany).

##### SD-OCT

Macular imaging was acquired using the Cirrus 5000 system (Carl Zeiss Meditech AG, Jena, Germany), including a 6 × 6 mm macular cube (512 × 128 A-scans) and high-definition five-line raster scans through the fovea.

##### FAF

Ultrawide retinal photography with the autofluorescence filter (532 nm excitation laser) was captured with the Optos California (Nikon, Tokyo, Japan). A color image of the fundus was also obtained for documentation.

##### MfERG

MfERG testing followed the 2021 International Society for Clinical Electrophysiology of Vision (ISCEV) standards.^19^ A 61-hexagon (m = 14) stimulus array with an on-luminance measured at 422 cd/m^2^ for a nominal 400 cd/m^2^ setting was presented using the Espion E3 system (Diagnosys LLC, Boston, MA, USA). Trial lenses containing the patient's habitual correction with the appropriate age-adjusted add power was placed in front of each eye.^20^ Recordings were obtained without dilation using monopolar Dawson–Trick–Litzkow corneal electrodes (DTLPlus; Diagnosys LLC). A gold disc electrode (Grass; Natus Manufacturing Limited, Galway, Ireland) on the center of the participant's forehead acted as the ground, and a gold disc electrode on each temple was the reference. Complete mfERG parameters are found in Supplementary Table S1. The mfERG stimulus settings, instrumentation, and testing protocol have not changed since the MERCI algorithm development study. When clinically suspected retinal pathology extended beyond the macula, a full-field electroretinogram (ffERG) was also completed.

An mfERG was obtained for every participant, whereas the other ancillary tests were completed in most cases with omissions occurring only when the participant left the appointment prematurely or an instrument was unavailable. All mfERG recordings were subjectively assessed for quality. Participants were excluded if severe ocular comorbidities markedly reduced mfERG amplitudes or if other noise sources resulted in uninterpretable waveforms such as poor patient performance. Remaining participants were divided into two groups: those who previously participated in the developmental study and provided data to train the MERCI algorithm (temporal dataset), and those newly acquainted to MERCI and consented during this study period (novel dataset).

#### Ground Truth

**Figure 1.**
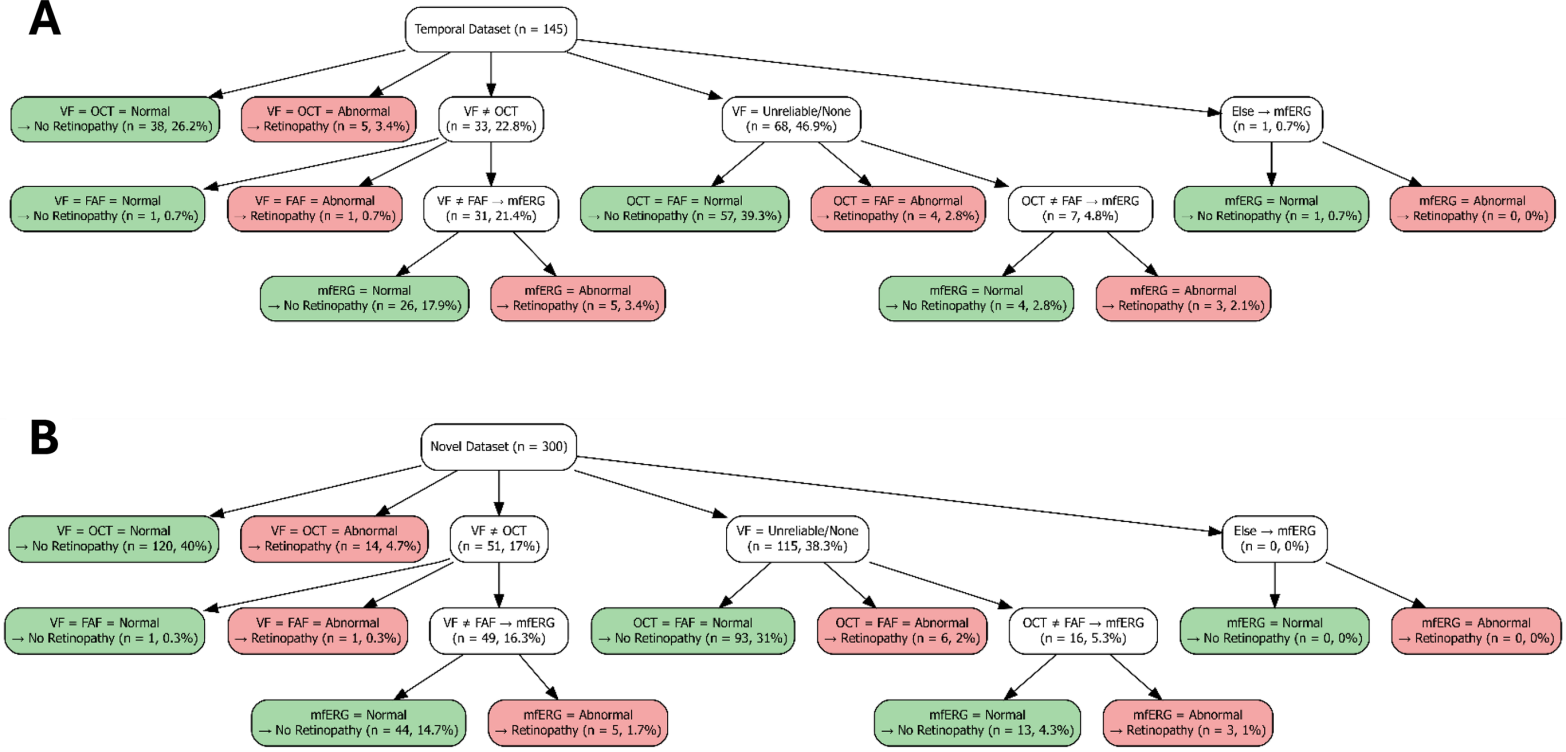
AAO HCQ Screening Guideline Decision Trees. (**A**) Temporal dataset. (**B**) Novel dataset. Percentages at each node are proportions of the total dataset.

A decision tree was developed to model the diagnostic reasoning used by ophthalmologists following AAO 2016 guidelines for identifying HCQ retinopathy. In accordance with guideline recommendations, automated perimetry was initially compared with SD-OCT, because of their wide clinical availability. In cases where SD-OCT and perimetry results were discordant, FAF was used as the subsequent objective modality, because a retinal fundus camera would likely be available in clinical practice. When VF results were deemed unreliable, FAF and SD-OCT findings were compared, which would be consistent with the first step in the RCOphth guidelines.^6^ If all prior comparisons remained inconclusive, the final determination of HCQ toxicity was based on the mfERG impression (Fig. 1).

Each diagnostic test from every participant was reviewed by G.W., and tests meeting quality criteria were examined for signs of HCQ toxicity. A subset of 100 images was independently reviewed by a retina specialist (G.M.) to assess interrater reliability. In cases where uncertainty arose, another retina specialist (B.G.B.) was consulted. Each test result was treated as independent, meaning it was not compared to the other tests from the same eye, nor was it compared to retrospective tests from the same patient.

#### Definitions of Toxicity

An abnormal FAF was defined as hyperautofluorescence or hypoautofluorescence particularly surrounding the parafovea. A pattern of pericentral toxicity was noted when abnormal autofluorescence was detected in the peripheral retina, beyond 8° from the fovea. Outer nuclear layer thinning or ellipsoid zone loss were signs of HCQ toxicity on SD-OCT.

VF were considered unreliable when fixation losses were equal to or greater than 20%, false positives or false negatives were equal to or greater than 33%, had artifacts (lid, lens rim), or glaucomatous field defects. Fields with five or more contiguous scotoma points were classified as abnormal.

MfERG were interpreted by a visual electrophysiologist (T.W.). Reductions in ring 1 wave amplitudes or ring ratios that exceeded clinic thresholds were annotated as abnormal.^18^ Clinic-specific normative values are found in Supplementary Table S2.

Participants were kept in the analysis even when their VF, OCT, or FAF showed abnormalities or artifacts, provided that the mfERG itself was not impacted. Because the mfERG was the last branch in the decision tree, it was crucial that a determinate outcome would result for each participant.

#### Analysis

After inspection of individual tests, results from both eyes for each test from the same participant were compared. The test was labeled abnormal if either eye showed an abnormal finding. Likewise, VF was classified as unreliable if one eye had an unreliable test. The resulting test labels were subsequently processed through the AAO decision tree to determine the participant's reference diagnosis.

The mfERG for each participant was analysed by the MERCI algorithm. Between the two eyes, the higher MERCI score was assigned to the participant. Using the MERCI score of 50 as the threshold for retinopathy, as dictated by Habib et al.^18^ in the development study, each participant was also labelled with a MERCI prediction.

MERCI predictions were compared against the corresponding AAO guideline-based reference diagnosis. Agreements in the prediction were labeled as true positives and true negatives, and discordances were assigned as false positives and false negatives. The sensitivity, specificity, positive predictive value (PPV), negative predictive value (NPV), accuracy, F1 and F2 score, and area under the receiver operator characteristic curve (AUROC) were calculated to represent the diagnostic accuracy of the algorithm.

#### Sensitivity Analyses

Subgroup analyses based on age, sex, reason for taking HCQ (i.e., disease), duration of HCQ treatment, and cumulative dose were conducted to assess model bias. Age was dichotomized at 60 years, treatment duration at 10 years, and cumulative dose at 1100 g. Disease was categorized as SLE, RA, or Other (Sjogren syndrome, mixed connective tissue disease, combination diagnosis, and other conditions). AUROC was calculated for each category to represent diagnostic ability.

An additional sensitivity analysis was performed removing cases where the decision tree outcome was based on the mfERG. Confusion matrices, performance metrics, and AUROC were recalculated.

### Data Analysis

Statistical modeling was conducted using R (v4.4.2; Bell Laboratories, Murray Hill, NJ, USA).^21^ Because of an imbalance of positive cases, interrater reliability was calculated using prevalence-adjusted bias-adjusted kappa (PABAK).^22^ Continuous variables were compared using the Student *t*-test or Mann-Whitney U test, and categorical variables using χ^2^ or Fisher exact tests. Statistical significance was *P* < 0.05. Sensitivity, specificity, PPV, NPV, and accuracy were reported with 95% confidence intervals (CIs) calculated using the Wilson score method.^23^ Confidence intervals for F1 and F2 scores were obtained using nonparametric bootstrap resampling (2000 iterations). The AUROC were compared using the DeLong test.^24^ Holm correction was used for multiple comparisons.

## Results

A total of 497 patients were recruited during the study period. Upon inspection of the mfERG recordings, 52 participants were excluded due to poor waveform morphology (*n* = 15) or due to findings suspicious for significant ocular comorbidities (*n* = 37). This resulted in 445 participants deemed suitable for further analysis. 145 participants overlapped with the MERCI developmental cohort, and their newly acquired test results comprised the temporal dataset. The remaining 300 newly consented participants constituted the novel dataset.

During two separate periods where the Humphrey Field Analyzer and the Optos were unavailable, only two participants (0.4%) did not undergo a VF and 20 participants (4.5%) did not complete fundus photography.

Interrater reliability between G.W. and G.M. was high for SD-OCT (PABAK = 0.94, 95% CI: 0.83–0.98; Cohen's κ = 0.62; 97% case agreement), FAF (PABAK = 0.78; 95% CI, 0.62–0.89; Cohen's κ = 0.25; 89% case agreement), and VF (PABAK = 0.84; 95% CI, 0.70–0.93; Cohen's κ = 0.93; 92% case agreement). Discrepancies in FAF interpretation occurred primarily as result of media-artifact and image quality.

Using the decision tree applied to the temporal dataset, 18 participants (12.4%) were classified as having HCQ retinopathy and 127 (87.6%) as not having HCQ retinopathy. In the novel dataset, 29 participants (9.7%) were classified as having HCQ retinopathy and 271 (90.3%) as not having HCQ retinopathy. In the temporal dataset, 29.6% of diagnostic decisions were resolved at the first decision node, where VF testing was concordant with SD-OCT findings. In contrast, 44.7% of decisions in the novel dataset were determined at this initial rule. When VF and SD-OCT findings were discordant, FAF served as the subsequent modality for comparison within the decision tree. Among these cases, concordance was rare. Only two cases demonstrated concordant VF and FAF findings in the absence of SD-OCT abnormalities, corresponding to 1.4% and 0.6% in the temporal and novel datasets respectively. The remaining VF to SD-OCT comparisons were discordant (21.4% temporal, 16.3% novel) and therefore required mfERG to reach a final determination. VF reliability differed between datasets. Unreliable VF results were observed in 46.9% of cases in the temporal dataset and 38.3% in the novel dataset, necessitating comparison between FAF and SD-OCT in these participants. Overall, mfERG was required to establish a diagnosis in 26.9% of all temporal dataset cases and 21.6% of the novel dataset cases.

Participants in the temporal dataset were on HCQ treatment significantly longer than those in the novel dataset, and as a result, these participants also had a higher cumulative dose. Conversely, they had a lower current weekly dose than those is in the novel dataset, because many participants reported that their rheumatologist had tapered their dosage to lower toxicity risk, once their autoimmune disease was under control (Table 1).

**Table 1.** Demographics and Hydroxychloroquine Treatment Details Stratified by Dataset

	Temporal Dataset	Novel Dataset	*P* Value
*N*	145	300	
Age, mean (SD)	59.28 (14.08)	57.16 (15.72)	0.17
Female sex	136 (93.8%)	263 (87.7%)	0.07
Disease			0.26^‡^
Combination	2 (1.4%)	11 (3.7%)	
Mixed connective tissue disease	0 (0.0%)	3 (1.0%)	
Other	6 (4.1%)	16 (5.3%)	
Rheumatoid arthritis	47 (32.4%)	116 (38.7%)	
Sjogren syndrome	2 (1.4%)	6 (2.0%)	
Systemic lupus erythematosus	88 (60.7%)	148 (49.3%)	
Duration (y), mean (SD)	16.57 (7.20)	12.48 (8.15)	<0.001^*^^,^^†^
Weekly Dose (mg), mean (SD)	1808.45 (572.23)	1946.10 (609.08)	0.04^*^^,^^†^
Cumulative Dose (g), mean (SD)	1556.19 (837.54)	1227.68 (880.14)	<0.001^*^^,^^†^
MERCI Score, mean (SD)	56.25 (45.12)	46.96 (46.38)	0.05^†^

* Significance.

† Mann Whitney U test.

‡ Fisher exact test.

Overall, people with HCQ toxicity were associated with older age (*P* = 0.04), longer treatment duration (*P* < 0.01), and higher cumulative dose (*P* < 0.01), factors known to be associated with increased risk for HCQ retinopathy (See Supplementary Table S3).^4^ Among HCQ retinopathy-negative participants, individuals in the temporal dataset (*n* = 127) had significantly longer HCQ exposure (16.69 ± 7.21 vs. 11.92 ± 8.00 years, *P* < 0.001) and higher cumulative doses (1557.57 ± 833.64 g vs. 1177.99 ± 872.30 g, *P* < 0.001) compared with those in the novel dataset (*n* = 271) (Table 2). However, weekly dose did not differ significantly. In contrast to participants classified as HCQ retinopathy-positive, no significant differences were observed between the temporal (*n* = 18) and novel (*n* = 29) datasets with respect to age, sex, disease distribution, duration of HCQ use, weekly dose, or cumulative dose (Table 2).

**Table 2.** Comparison of Demographics and HCQ Treatment Details Stratified by Toxicity and Dataset

	HCQ Retinopathy (−)			HCQ Retinopathy (+)		
	Temporal Dataset	Novel Dataset	*P* Value	Temporal Dataset	Novel Dataset	*P* Value
*N*	127	271		18	29	
Age, mean (SD)	58.80 (14.27)	56.65 (15.86)	0.17	62.72 (12.47)	61.93 (13.79)	0.84
Female sex	118 (92.9%)	238 (87.8%)	0.49	18 (100.0%)	25 (86.2%)	0.49
Disease			0.55^‡^			0.30^‡^
Combination	2 (1.6%)	9 (3.3%)		0 (0.0%)	2 (6.9%)	
Mixed connective tissue disease	0 (0.0%)	3 (1.1%)		0 (0.0%)	0 (0.0%)	
Other	6 (4.7%)	16 (5.9%)		0 (0.0%)	0 (0.0%)	
Rheumatoid arthritis	43 (33.9%)	105 (38.7%)		4 (22.2%)	11 (37.9%)	
Sjogren syndrome	2 (1.6%)	5 (1.8%)		0 (0.0%)	1 (3.4%)	
Systemic lupus erythematosus	74 (58.3%)	133 (49.1%)		14 (77.8%)	15 (51.7%)	
Duration (y), mean (SD)	16.69 (7.21)	11.92 (8.00)	<0.001^*^^,^^†^	15.69 (7.29)	17.86 (7.64)	0.29^†^
Weekly dose (mg), mean (SD)	1805.65 (570.59)	1947.00 (611.43)	0.06^†^	1827.78 (599.81)	1937.50 (597.00)	0.47^†^
Cumulative dose (g), mean (SD)	1557.57 (833.64)	1177.99 (872.30)	<0.001^*^^,^^†^	1546.71 (888.68)	1719.08 (817.82)	0.56^†^
MERCI score, mean (SD)	51.03 (45.64)	42.14 (45.71)	0.05^†^	93.06 (12.95)	92.03 (22.10)	0.81^†^

* Significance.

† Mann Whitney U test.

‡ Fisher exact test.

The negative retinopathy cases in the temporal dataset showed slightly higher mean MERCI scores compared with the novel dataset (51.03 ± 45.64 vs. 42.14 ± 45.71, *P* = 0.05), with the mean value marginally exceeding the threshold associated with toxicity. MERCI scores were similarly elevated in both groups of the HCQ positive cases, consistent with retinal dysfunction (93.06 ± 12.95 vs. 92.03 ± 22.10) (Table 2).

Within the temporal dataset, MERCI predicted toxicity from a mfERG in 55.8% (81/145) of cases, which was not significantly different from the 47.3% (142/300) of cases in the novel dataset (*P* = 0.10). However, when compared with the reference standard of the decision tree outcomes, these predictions represented 43.4% (63/145) and 38.3% (115/300) false positives. 44.1% (64/145) and 52.0% (156/300) of the temporal and novel datasets, respectively, were true negatives. False negatives were exceedingly rare, occurring in 0% (0/145) of the temporal dataset and only 0.7% (2/300) of the novel dataset (Table 3).

**Table 3.** Diagnostic Classification Outcomes Comparing MERCI Outputs (Algorithm Predictions) to the American Academy of Ophthalmology Hydroxychloroquine Screening Guideline Decision Tree Results (Reference Standard)

	Temporal Dataset (*n* = 145)	Novel Dataset (*n* = 300)
True positives	18 (12.4%)	27 (9.0%)
False positives	63 (43.4%)	115 (38.3%)
False negatives	0 (0.0%)	2 (0.7%)
True negatives	64 (44.1%)	156 (52.0%)

**Figure 2.**
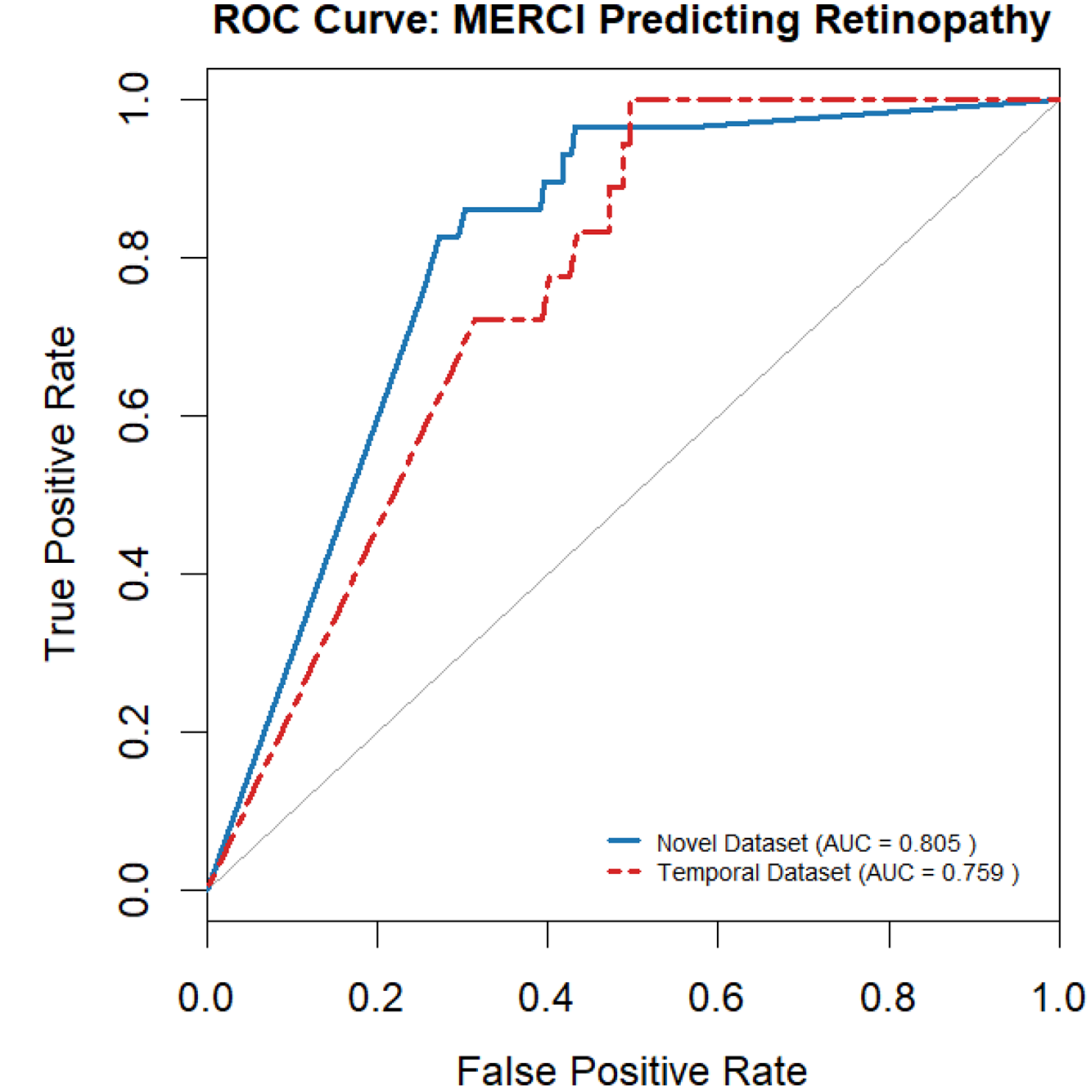
ROC curves for the novel (*solid blue line*) and temporal (*dashed red line*) datasets. Area under the curve (AUC) was calculated for the ROC curve for each dataset.

The predictive performance of MERCI using the temporal dataset achieved a sensitivity of 1.000 (95% CI, 0.824–1.000), specificity of 0.504 (95% CI, 0.418–0.589), PPV of 0.222 (95% CI, 0.145–0.324), NPV of 1.000 (95% CI, 0.943–1.000), accuracy of 0.566 (95% CI, 0.484–0.643), a F1 score of 0.364 (95% CI, 0.238–0.487), and a F2 score of 0.588 (95% CI, 0.438–0.700) (Table 4). Similar performance resulted in the novel dataset, reaching a sensitivity of 0.931 (95% CI, 0.780–0.981), specificity of 0.576 (95% CI, 0.516–0.633), PPV of 0.190 (95% CI, 0.134–0.263), NPV of 0.987 (95% CI, 0.955–0.997), accuracy of 0.610 (95% CI, 0.554–0.663), a F1 score of 0.316 (95% CI, 0.221–0.406), and a F2 score of 0.523 (95% CI, 0.406–0.619) (Table 4). Because of the comparable diagnostic accuracies between the two groups, the AUROC was also similar between the temporal and novel datasets (0.759 vs. 0.805, *P* = 0.38) (Fig. 2).

**Table 4.** External Validation Performance Metrics of the MERCI Algorithm (95% CI)

	Temporal Dataset	Novel Dataset	*P* Value
Sensitivity	1.000 (0.824–1.000)	0.931 (0.780–0.981)	
Specificity	0.504 (0.418–0.589)	0.576 (0.516–0.633)	
Positive predictive value	0.222 (0.145–0.324)	0.190 (0.134–0.263)	
Negative predictive value	1.000 (0.943–1.000)	0.987 (0.955–0.997)	
Accuracy	0.566 (0.484–0.643)	0.610 (0.554–0.663)	
F1 Score	0.364 (0.238–0.487)	0.316 (0.221–0.406)	
F2 Score	0.588 (0.438–0.700)	0.523 (0.406–0.619)	
AUROC	0.759 (0.678–0.841)	0.805 (0.744–0.867)	0.38

**Table 5.** Diagnostic Test Results for the False-Negative Cases

Subject ID	VF	OCT	FAF	mfERG	MERCI Score
3455	OD: Unreliable	OD: Normal	OD: Normal	OD: Normal	OD: 0
	OS: Normal	OS: Normal	OS: Abnormal	OS: Abnormal	OS: 46
	UNRELIABLE	NORMAL	ABNORMAL	ABNORMAL^*^	NORMAL
5967	OD: Normal	OD: Abnormal (pericentral)	OD: Abnormal (peripherally)	OD: Normal	OD: 0
	OS: Abnormal	OS: Abnormal: (pericentral)	OS: Abnormal (peripherally)	OS: Normal OU: ffERG reduced a-waves, b-waves close to lower limits	OS: 0
	ABNORMAL^*^	ABNORMAL^*^	ABNORMAL	NORMAL	NORMAL

^*^The node in the decision tree where HCQ retinopathy was determined.

AUROC in the subgroup analyses of age, sex, disease, duration and cumulative dose ranged from 0.732 to 0.837 (See Supplementary Figs. S1–S5). Comparisons of AUROC between subgroups did not result in significant differences (See Supplementary Tables S4–S8).

When datasets were reanalyzed without cases where the mfERG dictated the reference diagnosis, the performance metrics remained similar (See Supplementary Tables S9, S10). The AUROC for the mfERG-excluded temporal and novel datasets were 0.782 and 0.814 respectively, which was not significantly different from each other (*P* = 0.59), nor from their corresponding AUROC calculated from the mfERG-included datasets (*P* = 0.72 and *P* = 0.86, respectively) (See Supplementary Fig. S6, Supplementary Table S11).

### Case Analysis of False Negatives

Subject 3455 in the novel dataset was prescribed HCQ to treat SLE for12 years. She had normal OCTs but unreliable VFs. There was some evidence of hyperautofluorescence on the FAF in the left eye, but it was subtle because of low contrast caused by taking the photo through a small pupil. Since the OCT results were incongruous with first the VF and then also the FAF, the participant's reference label was determined by the mfERG. The mfERG of the right eye did not show signs of abnormality. However, the mfERG report concluded that this was a unilateral case of HCQ retinopathy because of amplitude reductions in the foveal and multiple localized regions of the parafovea in the left eye. The MERCI score was 0 and 46 for the right and left eye, respectively (Table 5).

Subject 5967, also from the novel dataset, had been on HCQ treatment for over 20 years for RA. The participant presented with peripheral visual field defects in the 10-2 VF and pericentral thinning of the outer retinal layers in the OCT. This concordance was sufficient to diagnose HCQ retinopathy according to the AAO decision tree guidelines. Further inspection of the FAF revealed circumferential hypoautofluorescence in the retinal periphery and confirmed a pericentral pattern of toxicity. However, the waveforms in the mfERG were normal, exhibiting amplitudes and ring ratios within the expected limits. MERCI was also unable to detect any abnormality in the mfERG, and assigned a score of 0 to both eyes. Because of the peripheral lesions, a ffERG was also performed. With scotopic stimulation, the a-waves and b-waves were reduced, and with photopic stimulation, the a-waves were preserved but the b-waves were below expected thresholds (Table 5).

### Discussion

We present an investigation into the stability of the MERCI algorithm in two datasets different from the one used to derive the model. The developmental study used a reference standard that defined toxicity if the mfERG exceeded clinic ring ratio thresholds and definite retinal changes to the macular OCT. In this study, we changed our annotation of toxicity to depend on a combination of diagnostic tests reflecting current clinic practice. Furthermore, individuals were assessed at a different scale, from the eye-level to a person-level. Despite not being trained on these factors, the diagnostic accuracy of MERCI remained high.

Consistent with both the temporal and novel datasets, MERCI achieved the highest metrics in sensitivity, and NPV. However, due to the high proportion of false positives, MERCI performed modestly in specificity and low in PPV. Considering these metrics, MERCI could be leveraged as a screening tool to rule out HCQ toxicity. Given that retinal damage from HCQ toxicity is irreversible, a screening modality with an emphasis on low false negatives is essential to prevent patients from continuing HCQ therapy that could lead to vision loss. Impressively, MERCI achieves less than 1% of false negatives in the datasets. A negative prediction by MERCI may suggest that no further testing would be required. Considering the costs of HCQ screening, in an analysis of following the 2011 version of the AAO guidelines, it was found that an estimated $33,155 to $344,172 (USD) per quality-adjusted life year is spent.^25^ This represents a 40% increase in costs compared to the 2002 edition of the guidelines, when screening only involved a VF, and no objective testing.^26^ Thus an HCQ retinopathy screening process that consists of fewer multimodal tests may reduce healthcare spending. If mfERG and MERCI analysis is implemented as an initial screen, assuming our reported true negative proportions, approximately 40% to 50% of patients could defer supplementary testing because screening would rely completely on the mfERG. A positive result, however, would necessitate additional testing to confirm the algorithm's prediction.

To improve MERCI, the algorithm could incorporate other inputs and biomarkers. At present, the SVM algorithm uses inputs of ring ratios, ring variation, and signal strength–all features of the mfERG only–to make a prediction. Kulyabin et al.^14^ trained two deep learning models to interpret full mfERG traces for HCQ toxicity, and reported superior performance compared to linear regression models. During development of MERCI, other machine learning models besides the SVM were not assessed. A future iteration of MERCI could use a more complex machine learning model, such as a neural network, to analyze complete mfERG waveforms and integrate HCQ treatment information like duration and dosage. Moving beyond a model based only on mfERG data, the AAO HCQ retinopathy screening decision tree is an algorithm of its own and could be used as the basis for a multimodal predictive model that includes VF, FAF, and OCT data. With such a model, SHAP analysis could help identify which diagnostic modalities and features contribute most strongly to diagnosing HCQ retinopathy.

MERCI appears to demonstrate stable performance when evaluated within the same environment and population as its training and development. On its own, mfERG offers greater sensitivity but reduced specificity relative to SD-OCT and VF individually.^27^ MERCI follows this trend, likely because it learns directly from mfERG waveforms, which are the sole input to the algorithm. As a result of MERCI's high sensitivity, it is responsive to small changes in mfERG waveform amplitudes and structure, thus classifying any mfERG with subtle deviations as HCQ retinopathy. This characteristic of MERCI may be beneficial if it is truly identifying early signs of HCQ retinopathy, but subject to error if these variations in mfERG responses are due to other ocular pathology or noise related to mfERG testing (e.g., dry eye, muscle artifact, poor fixation, loss of contact from the corneal electrode). To distinguish if the false positives are true errors or if they are individuals with early toxicity, analysis of longitudinal data would be required. Similarly, follow-up testing may clarify whether subject 3455 represents a valid false negative. MERCI assigned a borderline score to the left eye (46) and classified the right as completely normal (0). Because the value to the left eye is approaching threshold, it is possible that MERCI lacked just enough sensitivity to identify retinopathy in this instance. It is also important to note that the participant's reference diagnosis was based solely on mfERG findings, with amplitude reductions confined to the left eye. Given that HCQ retinopathy is typically bilateral and symmetric, and the reference standard relied on a single modality, the ground truth is uncertain. Repeat mfERG testing could resolve this, in which an increased MERCI score above threshold would support this case was a true error, whereas a stable or lower score would suggest signal variability rather than disease. A stronger case of external validation would involve evaluation of MERCI across varied populations and clinical settings, ideally within the intended end-user facilities.^28^ This would assess generalizability more rigorously. Additionally, although mfERG signal quality was assessed subjectively by an experienced electrophysiologist, inclusion of a standardized noise estimate in future studies may improve reproducibility and facilitate external validation across centers. Furthermore, the current MERCI score threshold to identify toxicity may be set too low, thereby prioritizing positive classifications to minimize false negatives and increasing false positives. Refinement of this threshold using larger and more diverse datasets, potentially tailored to specific populations, may improve performance.

The pericentral pattern of HCQ retinopathy is more frequently observed in individuals of East Asian ethnicity, although it can occur in non-Asian patients as well, albeit less commonly.^29^ Our study population was highly multicultural, and although race data were not collected, we did identify several patients with pericentral HCQ retinopathy. These patients had strikingly large patches of hypoautofluorescence in the region of the retinal arcades, and reduced waveforms in both their mfERG and ffERG. The MERCI algorithm is unable to classify patterns of retinopathy, but even in these pericentral cases, it was able to determine toxicity. It is possible that additional cases of pericentral retinopathy were present in our cohort but not recognized. Both the AAO and RCOphth screening guidelines recommend using 24-2 or 30-2 perimetry for Asian patients, so a wider visual field can be captured.^5^^,^^6^ More comprehensive detection of pericentral involvement could have been achieved using a 12 mm × 9 mm SD-OCT volume scan for East Asian patients.^30^ However, this was not feasible because the Cirrus OCT system used in our study does not support widefield imaging. If subtle changes occur outside of the 10-2 VF and the beyond the boundaries of the 6 × 6 mm OCT, the first node of the AAO decision tree will fail to recognize toxicity. It could be possible that some of the labeled false positives were indeed early detections of pericentral toxicity detected by MERCI. If true, this would indicate that MERCI's specificity is in fact underestimated and highlights the decision tree's deficiencies. Subject 5967 is an exception and reflects a true error made by MERCI. The participant had completely normal macular function as determined by the mfERG alone, but other ancillary testing supported a diagnosis of HCQ retinopathy. Retinal damage was in the far periphery—an atypical presentation of HCQ retinopathy, so it could be possible that MERCI cannot detect retinopathy beyond a certain degree of eccentricity.^31^

The delivery of the visual electrophysiology service needs to adapt to allow for more patients to access this technology.^32^ The limiting factor is the paucity of qualified interpreters. Automation through artificial intelligence and machine learning is one solution in filling in this gap.^33^ For example, in the inherited retinal disease space, a machine learning model was able to phenotype ABCA4 retinopathy from a ffERG.^34^ The mfERG may be de-emphasized in current HCQ retinopathy screening practices, given its position as the last node in the decision tree, yet nearly a quarter of cases remained indeterminate without it. Through MERCI's simplicity and automation, it has the potential to encourage broader use of the mfERG.

#### Limitations

A limitation was that a clinical diagnosis was not used as the ground truth. The visual electrophysiology lab at KVRC is a tertiary healthcare clinic that receives referrals from mainly the Greater Toronto Area, but also across the province of Ontario. Following ancillary testing, the results are returned to the referring ophthalmologist who integrates them with clinical findings to establish a diagnosis. Unless the referring doctor also practices at KVRC, the clinical diagnosis of patients is not typically returned to the electrophysiology lab. To ensure the research question could still be addressed of whether MERCI can predict toxicity as accurately as a clinician, the decision trees based on AAO HCQ retinopathy screening guidelines were constructed as a substitute. Although a diagnostic impression is not as robust, we believe that it is an acceptable alternative because it encapsulates multiple comparisons, even those beyond the original recommendations. A future study in collaboration with a practitioner who manages a high-volume HCQ patient population would facilitate access to clinical diagnoses and provide an opportunity to assess MERCI's real-world performance.

A further limitation was that a comparison with the developmental dataset could not be performed. Published sensitivity and specificity of MERCI were derived using ring ratio interpretation as the reference standard, which differs from the clinical reference used in this study.^18^ A direct comparison would require reanalysis of the developmental dataset using the same reference standard, involving application of AAO guideline–based decision trees to participants’ multimodal test results obtained at the time of assessment. However, during the creation of the MERCI algorithm, participants underwent only mfERG and SD-OCT testing. Considering both modalities are objective, and the AAO guidelines require confirmation with a subjective test, the decision tree does not contain branches that allow these two tests to be compared. Even if the two tests were compared, there would be no alternative rule to determine toxicity when the results did not come to a consensus. Although mfERG report impressions alone could have been used as the reference standard, both the AAO and RCOphth guidelines caution against relying on a single test, as this may be misleading.^5^^,^^6^ If metrics from MERCI's internal validation were available, it would allow assessment of potential overfitting. This would be observed as a decline in performance from higher development values, rather than remaining stable. However, the absence of performance degradation across subgroups, together with consistent results in validation using the temporal against the novel dataset, implies that MERCI is generalizable and lacks model bias. To better assess stability over time, a future intra-temporal validation study could instead use the results from the present study as the baseline rather than the development dataset.

We acknowledge that concordance bias was possible in this external validation, since approximately 27% of the temporal dataset and 22% of the novel dataset consisted of comparisons where the mfERG acted as both the prediction model input, and the reference label of HCQ retinopathy. To address this concern, a sensitivity analysis excluding cases where the mfERG impression dictated the reference diagnosis was conducted. The preservation of similar performance metrics in both datasets within these fully independent subsets supports the robustness of MERCI's diagnostic performance beyond this potential source of bias.

Last, the high sensitivity of the novel dataset, and notably the perfect sensitivity of the temporal dataset is based on small samples of ground truth positives. A single misidentified case by MERCI in the temporal dataset would turn one of the 18 true positive cases into a false negative, and considerably reduce the sensitivity from 1.000 to 0.944. The prevalence of HCQ retinopathy is low, so there is an inherent imbalance of patients who are HCQ retinopathy-negative than retinopathy-positive. By extending the study duration, more participants with HCQ retinopathy could be screened. This would be particularly advantageous in potentially capturing more patients rereferred for HCQ screening from the developmental dataset and increase the true positives of the temporal dataset, to verify its sensitivity.

## Conclusions

In this article, we performed a person-level external validation using a temporal and a novel dataset on the MERCI algorithm to confirm the reproducibility of its predictions and agreement with a multimodal diagnostic definition of toxicity. Because of its automation, and its high sensitivity and NPV, we believe that MERCI has potential as a first-line screening tool to rule out HCQ retinopathy.

## Supplementary Material

Supplement 1

Supplement 2
