# Supplementary material for: Validation of a Machine Learning Approach to the Analysis of Multifocal Electroretinograms for Hydroxychloroquine Retinopathy
Source: Transl Vis Sci Technol. 2026 Jul 28;15(7):29. doi: 10.1167/tvst.15.7.29 (PMC13426857; doi:10.1167/tvst.15.7.29)
Supplement: Supplement 1 [file tvst-15-7-29_s001.pdf]

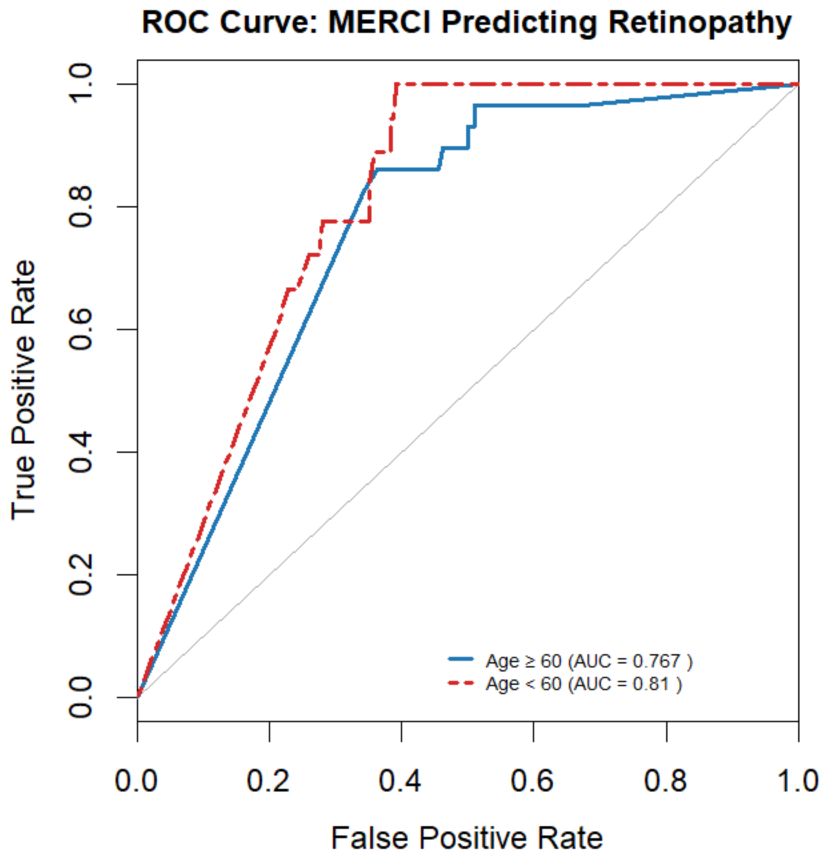

Figure S1. Receiver operating characteristic (ROC) curves stratified by age. The area under the curve (AUC) was calculated for participants aged ≥60 years (solid blue line) and <60 years (dashed red line).

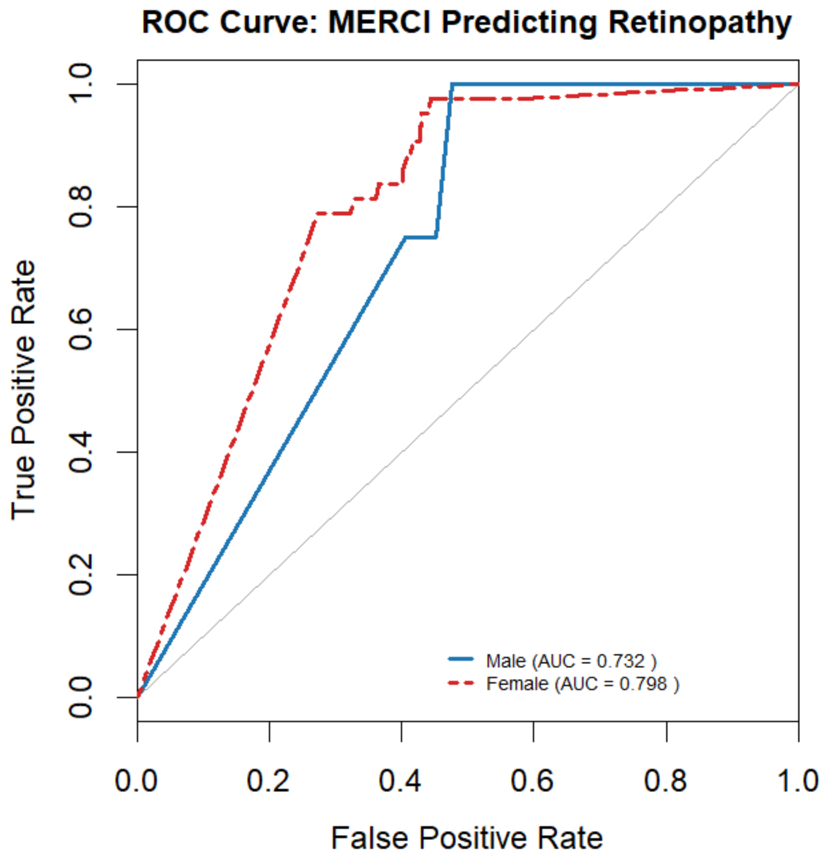

Figure S2. Receiver operating characteristic (ROC) curves stratified by sex. The area under the curve (AUC) was calculated for male (solid blue line) and female (dashed red line) participants.

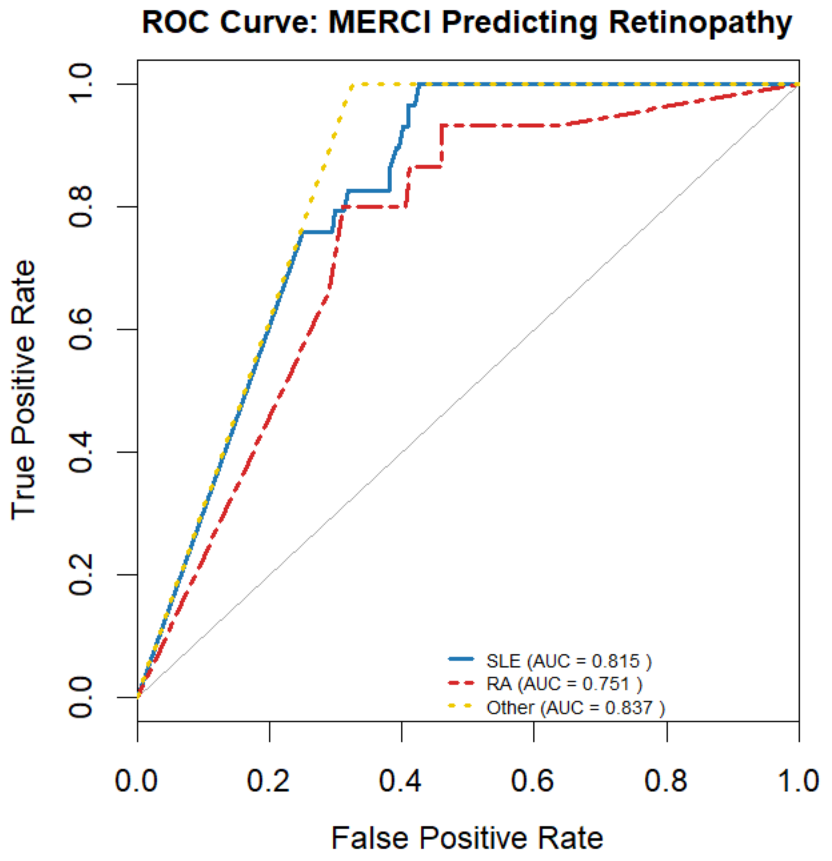

Figure S3. Receiver operating characteristic (ROC) curves stratified by disease. The area under the curve (AUC) was calculated for participants with systemic lupus erythematosus (SLE; solid blue line), rheumatoid arthritis (RA; dashed red line), and other autoimmune disease which includes Sjogren's syndrome, mixed connective tissue disease, or combinations of multiple diseases (Other; dotted yellow line).

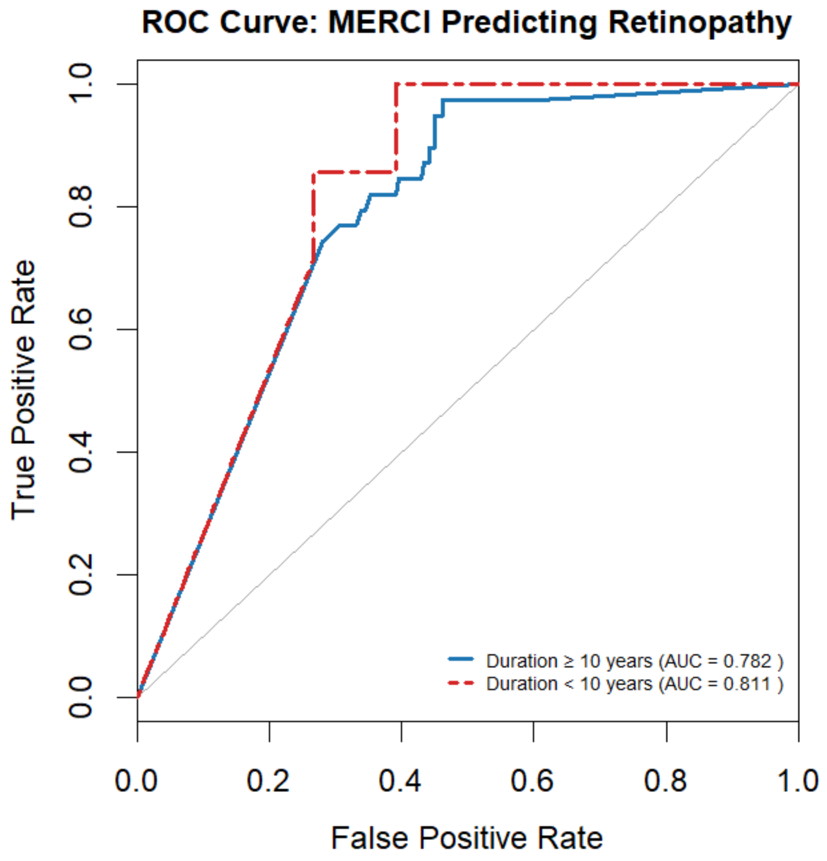

Figure S4. Receiver operating characteristic (ROC) curves stratified by hydroxychloroquine treatment duration. The area under the curve (AUC) was calculated for participants on treatment ≥10 years (solid blue line) and <10 years (dashed red line).

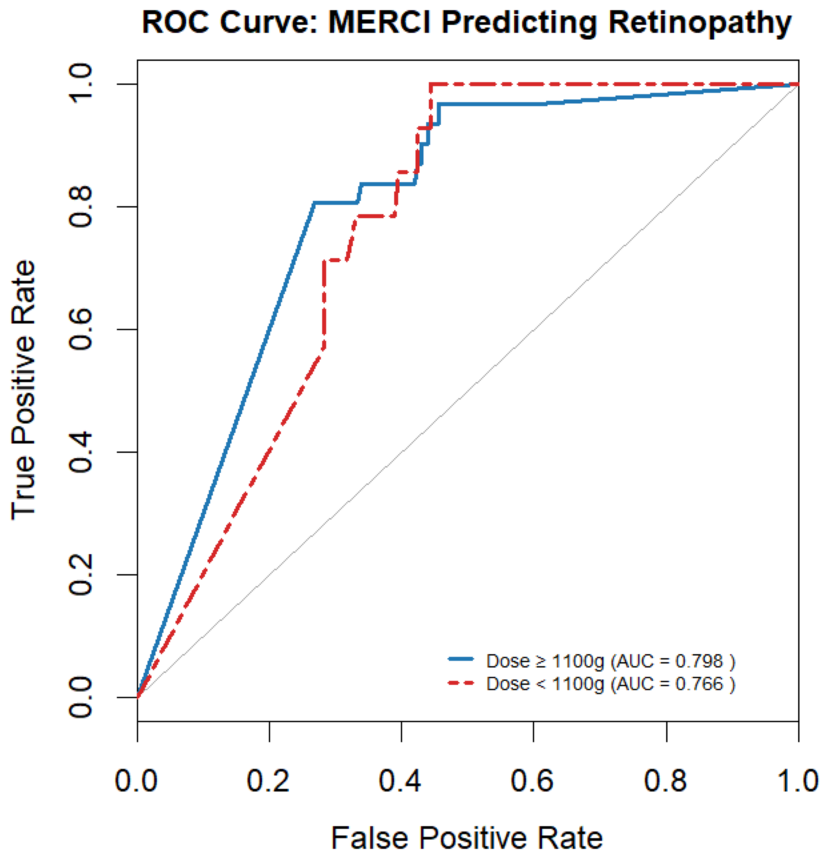

Figure S5. Receiver operating characteristic (ROC) curves stratified by hydroxychloroquine cumulative dose. The area under the curve (AUC) was calculated for dose ≥1100g (solid blue line) and <1100g (dashed red line).

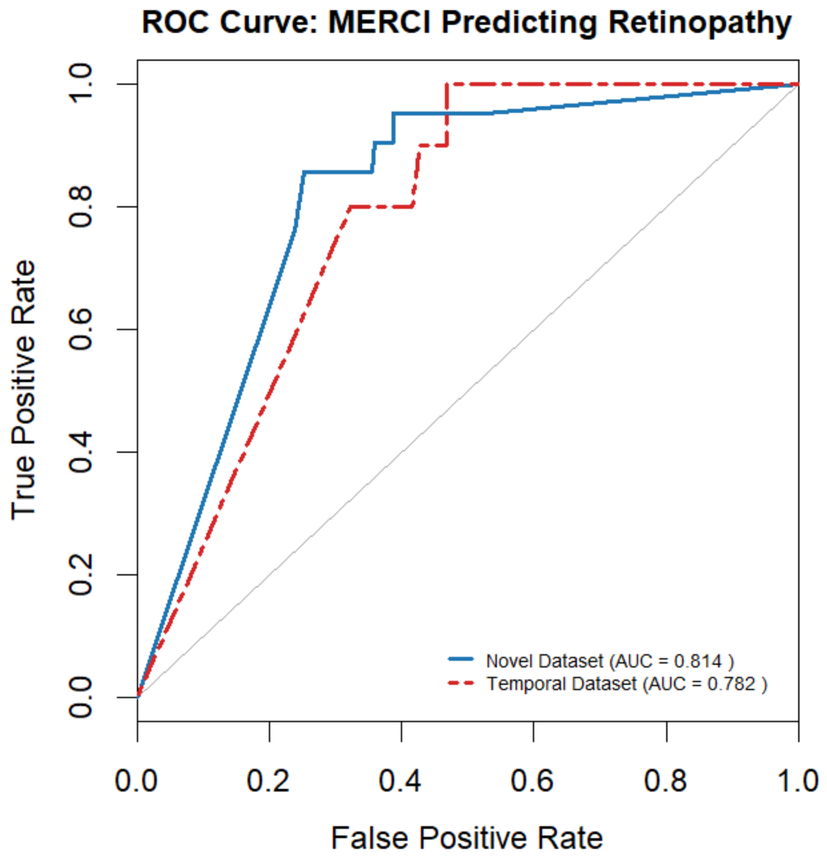

Figure S6. Receiver operating characteristic (ROC) curves for the novel (solid blue line) and temporal (dashed red line) datasets without the cases where the multifocal electroretinogram determined the reference label. Area under the curve (AUC) was calculated for the ROC curve for each dataset.
