# Supplementary material for: Validation of a Machine Learning Approach to the Analysis of Multifocal Electroretinograms for Hydroxychloroquine Retinopathy
Source: Transl Vis Sci Technol. 2026 Jul 28;15(7):29. doi: 10.1167/tvst.15.7.29 (PMC13426857; doi:10.1167/tvst.15.7.29)
Supplement: Supplement 2 [file tvst-15-7-29_s002.docx]

| **Table S1. Multifocal Electroretinogram Test Parameters** | | | | | |
| --- | --- | --- | --- | --- | --- |
| **View** | Retinal (TN) | **Correlated** | 80 ms | **Colour On** | White |
| **Hexagons** | 61 | **Sequence Bits** | 14 | **Luminance On** | 422 cd/m^2^ |
| **Scaled** | True | **Smoothing** | Average [1] | **Colour Off** | Black |
| **Distortion** | 1-3.2 | **Filtering** | FFT [4] | **Luminance Off** | 0 cd/m^2^ |
| **Filter** | 10-100 Hz | **Filler Frames** | 0 | **Mains Rejection** | Off |
| **Base Period** | 13.3 ms | **Electrodes** | DTL Fiber | **Noise Rejection** | 1 passes |
| **Kernel Order** | 1 | **Background** | Mean Luminance | **Markers Relative** | P1-N1 |
| **Number of Segments** | 8 | **Fixation Size** | 5.00 |  |  |
| **Segment Length** | 30 sec | **Fixation Width** | 0.20 |  |  |

| **Table S2. Clinic-Specific Multifocal Electroretinogram Ring Ratio Thresholds** | | | | | | |
| --- | --- | --- | --- | --- | --- | --- |
|  | **Lower**  **Threshold** | | | | | **Upper Threshold** |
| **Age** | **20-29** | **30-39** | **40-49** | **50-59** | **60+** | **All** |
| **R_1_** | 14.9 | 12.7 | 10.5 | 8.3 | 6.1 |  |
| **R_1_/R_2_** |  |  |  |  |  | 2.7 |
| **R_1_/R_3_** |  |  |  |  |  | 5.5 |
| **R_1_/R_4_** |  |  |  |  |  | 8.4 |
| **R_1_/R_5_** |  |  |  |  |  | 12.6 |

| **Table S3. Demographics and Hydroxychloroquine (HCQ) Treatment Details Stratified by Toxicity** | | | |
| --- | --- | --- | --- |
|  | **HCQ Retinopathy -** | **HCQ Retinopathy +** | **p-value** |
| **n** | 398 | 47 |  |
| **Age (mean (SD))** | 57.33 (15.38) | 62.23 (13.17) | 0.04* |
| **Female sex (n (%))** | 356 (89.4) | 43 (91.5) | 0.86 |
| **Disease (%)** |  |  | 0.40^‡^ |
| **Combination** | 11 (2.8) | 2 (4.3) |  |
| **Mixed Connective Tissue Disease** | 3 (0.8) | 0 (0.0) |  |
| **Other** | 22 (5.5) | 0 (0.0) |  |
| **Rheumatoid Arthritis** | 148 (37.2) | 15 (31.9) |  |
| **Sjogren Syndrome** | 7 (1.8) | 1 (2.1) |  |
| **Systemic Lupus Erythematosus** | 207 (52.0) | 29 (61.7) |  |
| **Duration (y) (mean (SD))** | 13.45 (8.06) | 17.01 (7.50) | <0.01*^†^ |
| **Weekly Dose (mg) (mean (SD))** | 1902.17 (601.67) | 1894.57 (593.88) | 0.70^†^ |
| **Cumulative Dose (g) (mean (SD))** | 1298.37 (877.18) | 1650.13 (841.22) | <0.01*^†^ |
| **MERCI Score (mean (SD))** | 44.98 (45.82) | 92.43 (18.96) | <0.001*^†^ |
| * = Significance, † = Mann Whitney U, ‡ = Fisher | | | |
| **Table S4. Area Under the Receiver-Operating Characteristic Curve (95% CI) with Subgroup Analysis Based on Age** | | | |
| **Age ≥ 60** | **Age < 60** | **p-value** | |
| 0.767  (0.699 – 0.834) | 0.810  (0.746 – 0.875) | 0.36 | |

| **Table S5. Area Under the Receiver-Operating Characteristic Curve (95% CI) with Subgroup Analysis Based on Sex** | | |
| --- | --- | --- |
| **Female** | **Male** | **p-value** |
| 0.798  (0.748 – 0.849) | 0.732  (0.574 – 0.890) | 0.44 |

| **Table S6. Area Under the Receiver-Operating Characteristic Curve (95% CI) with Subgroup Analysis Based on Disease** | | | |
| --- | --- | --- | --- |
| **Systemic Lupus Erythematosus (SLE)** | **Rheumatoid Arthritis (RA)** | **Other** | **p-value** |
| 0.815  (0.761 – 0.868) | 0.751  (0.645 – 0.856) | 0.837  (0.766 – 0.908) | SLE vs. RA: 0.58  SLE vs. Other: 0.62  RA vs. Other: 0.55 |

| **Table S7. Area Under the Receiver-Operating Characteristic Curve (95% CI) with Subgroup Analysis Based on Duration** | | |
| --- | --- | --- |
| **Duration ≥ 10 Years** | **Duration < 10 Years** | **p-value** |
| 0.782  (0.726 – 0.839) | 0.811  (0.721 – 0.900) | 0.60 |

| **Table S8. Area Under the Receiver-Operating Characteristic Curve (95% CI) with Subgroup Analysis Based on Cumulative Dose** | | |
| --- | --- | --- |
| **Dose ≥ 1100g** | **Dose < 1100g** | **p-value** |
| 0.798  (0.734 – 0.863) | 0.766  (0.690 – 0.841) | 0.52 |

| **Table S9. Diagnostic Classification Outcomes Comparing MERCI Outputs (Algorithm Predictions) to the American Academy of Ophthalmology Hydroxychloroquine Screening Guideline Decision Tree Results (Reference Standard) Without the Cases where the Multifocal Electroretinogram Determined the Reference Label** | | |
| --- | --- | --- |
|  | **Temporal Dataset (n = 106)** | **Novel Dataset (n = 235)** |
| **True Positives** | 10 (9.4%) | 20 (8.5%) |
| **False Positives** | 46 (43.4%) | 85 (36.2%) |
| **False Negatives** | 0 (0.0%) | 1 (0.4%) |
| **True Negatives** | 50 (47.2%) | 129 (54.9%) |

| **Table S10. External Validation Performance Metrics of the MERCI Algorithm (95% CI) Without the Cases where the Multifocal Electroretinogram Determined the Reference Label** | | |
| --- | --- | --- |
|  | **Temporal Dataset** | **Novel Dataset** |
| **Sensitivity** | 1.000  (0.722 – 1.000) | 0.952  (0.773 – 0.992) |
| **Specificity** | 0.521  (0.422 – 0.618) | 0.603  (0.536 – 0.666) |
| **Positive Predictive Value** | 0.179  (0.100 – 0.298) | 0.190  (0.127 – 0.276) |
| **Negative Predictive Value** | 1.000  (0.929 – 1.000) | 0.992  (0.958 – 0.999) |
| **Accuracy** | 0.566  (0.471 – 0.656) | 0.634  (0.571 – 0.693) |
| **F1 Score** | 0.303  (0.161 – 0.448) | 0.317  (0.214 – 0.421) |
| **F2 Score** | 0.521  (0.320 – 0.672) | 0.529  (0.387 – 0.640) |

| **Table S11. Comparison of Areas Under the Receiver Operator Characteristic Curves Between Datasets** | | | |
| --- | --- | --- | --- |
|  | **Temporal Dataset** | **Novel Dataset** | **p-value** |
| **mfERG Included** | 0.759  (0.678 – 0.841) | 0.805  (0.744 – 0.867) | 0.38 |
| **mfERG Excluded** | 0.782  (0.690 – 0.874) | 0.814  (0.740 – 0.888) | 0.59 |
| **p-value** | 0.72 | 0.86 |  |
